# Ingested Foreign Body Migration to the Liver: An Unusual Cause of Persistent Abdominal Pain in a 54-Year-Old Female

**DOI:** 10.1155/2018/8745271

**Published:** 2018-01-21

**Authors:** Alan Lucerna, James Espinosa, Kelly Schuitema, Risha Hertz

**Affiliations:** ^1^Emergency Medicine, Rowan University SOM/Jefferson Health, Stratford, NJ, USA; ^2^OMS 2, Philadelphia College of Osteopathic Medicine, Philadelphia, PA, USA; ^3^Department of Family Medicine, University of Pennsylvania/Penn Medicine, Gibbsboro, NJ, USA

## Abstract

Abdominal pain is a common presentation in emergency medicine. We describe the case of a 54-year-old female who presented to the emergency department due to worsening abdominal pain. She had a history of right upper quadrant (RUQ) abdominal pain that had been ongoing for several months. The pain had been thought by the primary care team to be related to gastritis and she had been prescribed a proton pump inhibitor (PPI). Her abdominal pain increased in the three days prior to her presentation to the emergency department (ED). The computed tomography (CT) scan of the abdomen showed a foreign body (FB) in the liver which was successfully removed surgically. Pathology results showed that the FB was consistent with a small bone fragment. Ingestions of FB are common but seldom result in complications. When complications do arise, perforation of a hollow viscous is typically seen. Rarely, transmigration of the FB can occur.

## 1. Introduction

Acute abdominal pain, which accounts for 7–10% of all ED visits, is defined as pain of nontraumatic origin with a maximum duration of 5 days [1]. About 40% of abdominal pain encountered in the ED is of uncertain cause [2]. Here we describe a case of a hepatic FB in a patient with right upper quadrant (RUQ) pain that had been ongoing for several months. Ingestions of FBs are common. Most of the patients do not recall the ingestion [3]. Rarely complications can occur and when they do arise, perforation of a hollow viscous can be seen. Transmigration of the FB can also occur. Complications from a transmigration of an FB from the intestines to the liver may present months to even years after the ingestion [4].

## 2. Case Report

A 54-year-old female presented to the ED with a complaint of RUQ abdominal pain which was aching in nature, was constant, and had been present for several months. The pain had been thought by the primary care team to be related to gastritis. She had been prescribed a proton pump inhibitor (PPI). Her abdominal pain increased in the three days prior to her presentation to the ED. She denied weight loss. There was no history of hematochezia or stool changes. She denied chest pain, shortness of breath, or fevers. She did not take any nonsteroidal anti-inflammatory drugs (NSAIDs) and she had no history of peptic ulcer disease (PUD).

On presentation, her vital signs were blood pressure 145/88 mm/Hg, heart rate 109 beats per minute, respiratory rate 18 breaths per minute, temperature 98.2 degrees Fahrenheit orally, and a pulse oximetry of 100% on room air. Her Body Mass Index (BMI) was 24.02 kg/m^2^.

Physical examination revealed tenderness to palpation on the RUQ of the abdomen but was otherwise unremarkable.

The patient's white blood cell count (WBC) was 11 × 10*∗*3/uL, with a hemoglobin of 10.4 g/dL and a platelet count of 327 × 10*∗*3/uL. Her alkaline phosphatase was mildly elevated (127 U/L) but her ALT and AST were within normal limits.

Given the location of her pain, she was sent for a RUQ ultrasound which was unremarkable. The chest X-ray was unremarkable. An electrocardiogram was done (ECG) which did not show any ischemic changes.

**Figure 1:**
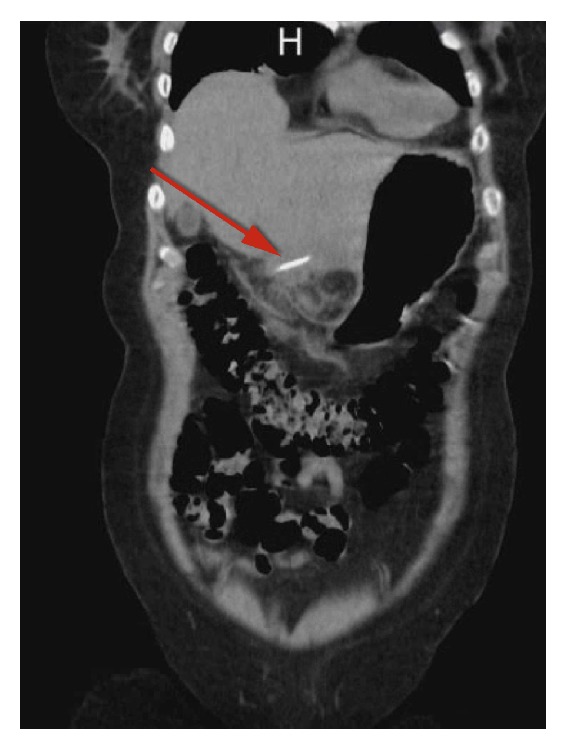
Coronal view CT scan of the abdomen and pelvis showing radiopaque FB in the liver (red arrow).

**Figure 2:**
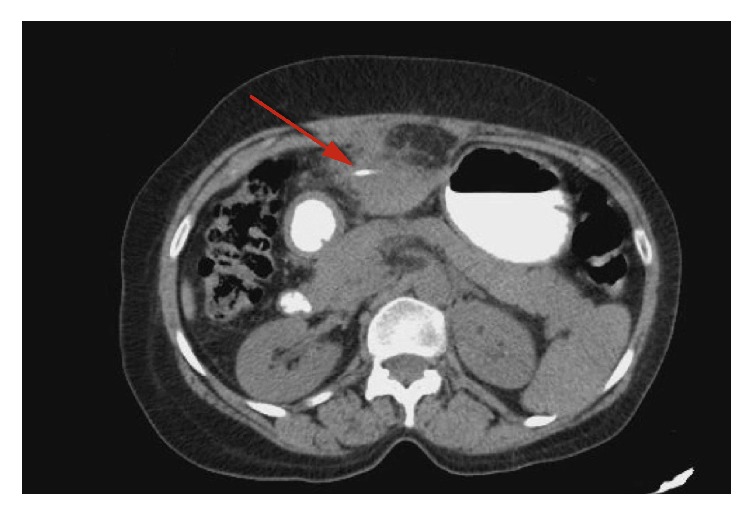
Axial view CT scan of the abdomen and pelvis showing a radiopaque FB in the liver (red arrow).

A CT scan of the abdomen and pelvis with oral contrast showed a linear radiodense FB along the left lobe of the liver with surrounding inflammatory changes which extended toward the body of the stomach with no evidence of free air (Figures 1 and 2).

After consultation with the surgical service, the patient was taken to the operating room for a diagnostic laparoscopy and removal of the FB. The FB was successfully removed and was noted to resemble a toothpick. The pathology report however identified the foreign object as a nonmetallic, greyish-white, hard, elongated, material measuring 1.6 × 0.2 × 0.1 cm, consistent with an FB comprising bone.

After 4 days in the hospital, the patient's pain resolved and she was discharged home.

## 3. Discussion

Ingested Fbs can be quite diverse in nature. Ingestions of dentures, fish bones, chicken bones, toothpicks, and cocktail sticks have been described [3]. Most of these FBs do not cause any serious complications. Typically, the FB passes through the gastrointestinal tract after one week with no complications [4–6]. Less than 1% of FB ingestions cause intestinal perforations [5]. Some patients do not recall the inciting ingestion [3]. Complications of a FB may take months to even years to manifest [4]. Interestingly, complications of a FB can also mimic appendicitis and diverticulitis [5].

Coyte et al. reported risk factors of FB ingestions and complications. Dentures are a risk factor. Alcoholism, mental illness, and intestinal strictures are also risk factors [5].

Patients with FB transmigration to the liver may present with abdominal pain, jaundice, fevers, liver function test abnormalities, vomiting, anorexia, or weight loss [6, 7]. Most are asymptomatic [6].

When FBs migrate outside the GI tract, patients may present with nonspecific symptoms [4]. The most common complication below the diaphragm is perforation. Owing to their narrow lumen and high angulation, the terminal ileum and the rectosigmoid junction are the usual sites of perforation [5]. Transmigration through the duodenum has also been reported [7].

However, even in the event of a perforation, free air is seldom seen. The site of perforation may be closed off by the resulting inflammatory changes or by the omentum. Because of these mechanisms, such perforations rarely lead to peritonitis [4, 5].

Migration of a FB to the liver is rare and typically results from penetration of the lumen of the stomach, duodenum, or transverse colon [6]. According to Santos et al., the most common site of perforation to the liver is through the stomach with the most common FB being fish bones [7]. Hepatic abscesses rarely result. The left lobe of the liver is more often affected than the right side [7].

Plain X-rays are only 32% sensitive in detecting FBs in the upper digestive tract. A radiodense FB may be obscured by fluids and solid organs. Additionally, the FB may not be sufficiently radiodense to be detected [4].

Ultrasound (US) can be used as an imaging modality. However, detection can be limited by a large body habitus or overlying bowel gas. The RUQ US in our patient did not detect any FB.

CT scan is therefore recommended. CT can detect the migration of an FB outside of the digestive tract and can identify such complication as perforation and abscess [4].

Oral contrast may obscure the faint calcification of the FB. Additionally, in CT scans where IV contrast is utilized, the FB may mimic blood vessels [4]. Coronal and sagittal views of the CT scan should be reviewed as the axial orientation of the FB may affect detection.

Once the FB is identified, surgery is typically performed to remove the object, as in our case. Hepatic abscesses when present are treated with drainage and antibiotics [7].

## 4. Conclusions

We describe the case of a FB found in the liver of a patient with RUQ pain for months. Ingestion of a FB is common but complications are rare and may include hollow viscous perforation and transmigration. These complications may occur distant to the initial ingestion. Because the perforation site is often sealed off by inflammatory changes or by the omentum, patients typically present without peritoneal signs.
